# Exosomes and their cargo proteins in diagnosis, process and treatment of gastric cancer

**DOI:** 10.3389/fcell.2025.1560583

**Published:** 2025-07-10

**Authors:** Wenjing Lu, Minghan Li, DanZeng LaMu, Hui Qian, Zhaofeng Liang, Xuezhong Xu

**Affiliations:** ^1^ Wujin Institute of Molecular Diagnostics and Precision Cancer Medicine of Jiangsu University, Wujin Hospital Affiliated With Jiangsu University, Changzhou, Jiangsu, China; ^2^ Jiangsu Key Laboratory of Medical Science and Laboratory Medicine, School of Medicine, Jiangsu University, Zhenjiang, Jiangsu, China

**Keywords:** exosomes, proteins, diagnosis, occurrence, development, treatment, gastric cancer

## Abstract

Gastric cancer is one of the common malignant tumors of digestive tract. Early diagnosis, process monitoring, and appropriate treatment strategies are crucial to reducing mortality and improving patient outcomes. However, the lack of specific early symptoms and reliable diagnostic markers often leads to delayed diagnosis and suboptimal treatment strategies. Exosomes, as small vesicular structures derived from endosomes, play crucial roles in cell-to-cell communication and have emerged as promising biomarkers and therapeutic targets in various cancers, including gastric cancer. This comprehensive review delves into the significance of exosomes and their cargo proteins, particularly focusing on their applications in the diagnosis, progress and treatment of gastric cancer. Based on this review, we believe that the real-time release characteristics of extracellular vesicle proteins make them an ideal tool for dynamically monitoring gastric cancer progression and treatment response. The potential of extracellular vesicles in “liquid biopsy” can be explored to replace traditional invasive examinations and achieve non-invasive and continuous disease monitoring. In the future, nanotechnology can be combined with artificial intelligence to develop an efficient extracellular vesicle protein capture and analysis platform, in order to enhance diagnostic sensitivity and specificity.

## 1 Introduction

Gastric cancer ranks among the leading causes of cancer-related deaths globally, with an estimated 1 million new cases diagnosed annually and about 769,000 deaths in 2020 (Tong et al., 2024; Lu et al., 2023). Gastric cancer remains a significant health burden globally, with high mortality rates despite advancements in treatment. Early diagnosis, process monitoring, and appropriate treatment strategies are crucial to reducing mortality and improving patient outcomes (Zhou et al., 2024). At present, the diagnostic methods and process detection of gastric cancer lack sensitivity and specificity. However, the lack of specific early symptoms and reliable diagnostic markers often leads to delayed diagnosis, with most gastric cancer patients presenting in advanced stages (Guan et al., 2023). Therefore, there is an urgent need to explore new, sensitive, and specific diagnostic biomarkers and process detection biomarkers, as well as new therapeutic targets (Chen et al., 2019; Cassotta et al., 2023; Afrin et al., 2020).

Exosomes, small membrane-bound vesicles derived from various cell types, are secreted into bodily fluids such as blood, urine, ascites, and saliva (Yu et al., 2022; Cianciosi et al., 2022). Exosomes carry a diverse array of biomolecular cargo, including nucleic acids, proteins, lipids, enzymes, and metabolites that reflect the physiological state of their parental cells (Yu et al., 2022; Lu et al., 2021; Liang et al., 2023; Chen et al., 2024). In the context of cancer, tumor-derived exosomes release substances that mirror the characteristics of the parental tumor cells, positioning them as potential diagnostic biomarkers for cancer. Exosomes play pivotal roles in different stages of tumor progression, including tumor-associated immune regulation, microenvironment remodeling, angiogenesis, epithelial-mesenchymal transition (EMT), invasion, and metastasis. Notably, the protein expression profiles of exosomes vary significantly across different types and stages of cancer, indicating their close association with cancer development and progression (Hyung et al., 2023; Dolatshahi et al., 2024). This review explores the role of exosomes and their cargo molecules in gastric cancer diagnosis and treatment.

## 2 Exosome biology and characteristics

Exosomes are naturally occurring, disk-shaped vesicles secreted by various cell types into the extracellular environment (Xu et al., 2024). They belong to a broader category of extracellular vehicles (EVs), which can be broadly divided into two main types: ectosomes and exosomes. Ectosomes are released through outward budding of the plasma membrane and range in size from 50 nm to 1 mm (Xu et al., 2024). In contrast, exosomes are derived from endosomes and have a diameter ranging from approximately 30–150 nm (Kalluri and LeBleu, 2020; Fu et al., 2019).

The biogenesis of exosomes involves the inward budding of the cell membrane, encapsulating extracellular components and membrane proteins to form early sorting endosomes (ESEs) (Xu et al., 2024). These ESEs can undergo material exchange with other organelles or fuse with each other to form late sorting endosomes (LSEs), which further develop into multivesicular bodies (MVBs) (Li B. et al., 2024). These MVBs contain numerous intraluminal vesicles (ILVs), which eventually be released as exosomes upon fusion with the plasma membrane (Chen et al., 2022; Moyano et al., 2019). Alternatively, MVBs can fuse with lysosomes or autophagosomes for degradation.

**FIGURE 1:**
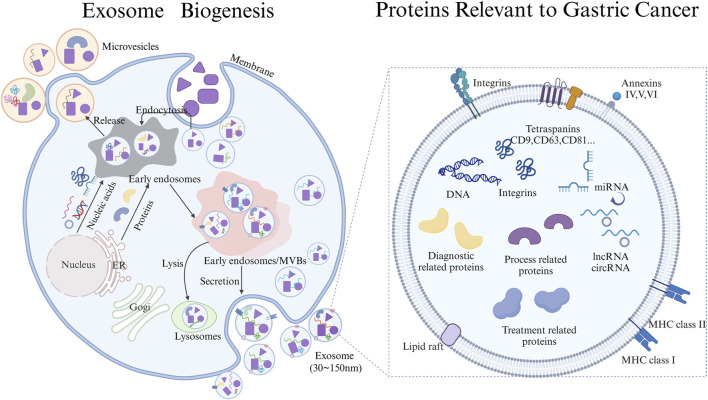
Schematic Diagram of Exosome biogenesis and proteins carried Relevant to the diagnosis, progression, and treatment of gastric cancer draw through Biorender.

Exosomes are secreted by various cell types and can be detected in various body fluids, including blood, urine, ascites, and saliva. Exosomes contain a complex cargo of biomolecules, including nucleic acids, proteins, lipids, enzymes, and metabolites, which reflect the physiological state of their parent cells. In addition to their heterogeneity, exosomes play crucial roles in various physiological processes, including immune responses, antigen presentation, immune evasion, and tumor invasion (Xu et al., 2024; Liang et al., 2022; Wu et al., 2021). They can be harnessed for immune modulation and cancer diagnosis and treatment. However, the specific role of exosomes and their proteins in early diagnosis, process monitoring, and treatment strategy selection of gastric cancer is not fully understood. Clarifying the role exosomes and their proteins in these processes is crucial for effective treatment and diagnostic applications of gastric cancer (Figure 1).

## 3 Role of exosomes and their cargo proteins in gastric cancer diagnosis

Exosomes have emerged as promising biomarkers for gastric cancer diagnosis due to their ability to carry tumor-specific bioactive components. Several studies have demonstrated that exosomal proteins, miRNAs, lncRNAs, and circRNAs are upregulated in gastric cancer patients compared to healthy controls, suggesting their potential as diagnostic biomarkers (Wu et al., 2021; Zhang et al., 2023). For instance, exosomal MT1-MMP mRNA has been found to be elevated in gastric cancer patients and is significantly correlated with tumor metastasis and TNM stage (Dong et al., 2019). The authors evaluated the diagnostic value of CEA, CA19-9, and CA72-4 in patients with gastric cancer, healthy controls, and patients with chronic gastritis or atypical hyperplasia, and found that only CEA exhibited significant differences. The area under the curve (AUC) for exosomal MT1-MMP was 0.788, which surpassed that of CEA at 0.655. When both markers were combined, the AUC increased to 0.821. Therefore, combinations of exosomal biomarkers with traditional serum tumor markers, such as CEA, CA19-9, and CA72-4, have shown improved diagnostic accuracy (Dong et al., 2019).

Cancer-associated fibroblast-derived exosomal DACT3-AS1 was a suppressive regulator in malignant transformation and ferroptosis-mediated oxaliplatin resistance in gastric cancer (Qu et al., 2023). DACT3-AS1 could be used for diagnosis and treatment of gastric cancer. It is reported that plasma exosome proteins ILK1 and CD14 were correlated with organ-specific metastasis in patients with advanced gastric cancer (Zhou et al., 2023). Peritoneal metastasis frequently occurs in patients with gastric cancer and represents a primary cause of mortality. Therefore, effective diagnosis of peritoneal metastasis in patients with gastric cancer is conducive to taking reasonable treatment measures. Proteomic profiling of gastric cancer with peritoneal metastasis revealed proteins (DUOXA2, ITGA7, LIMS1, MSRB3, PLCB1, RAB6B, SEMA3C, SMTN, TADA1, and TBC1D14) protein signature linked to the immune microenvironment and patient outcome, which were enriched in exosomes and cell adhesion pathways and may play an important role in the diagnosis of gastric cancer peritoneal metastasis (Chen et al., 2023). Identifying HER2 in serum-derived exosomes from advanced gastric cancer patients represented a promising biomarker for assessing tissue HER2 status and predicting the efficacy of trastuzumab-based therapy (Li Q. et al., 2023).

The results of Li et al. demonstrated that exosomal PD-L1 has the potential to become a prognostic and diagnostic biomarker for gastric cancer patients (Li H. et al., 2024). The findings presented by Fu et al. indicated that exosomal TRIM3 could potentially serve as a biomarker for the diagnosis of gastric cancer and the delivery of TRIM3 via exosomes may offered a novel approach for the treatment of gastric cancer (Fu et al., 2018). It is reported that the exosome-delivered Frizzled 10 may be a promising novel diagnostic and prognostic biomarker for gastric cancer (Scavo et al., 2021). Exosome analysis validated the expression of ITIH4 in the sera of gastric cancer patients, whereas it was absent in the sera of healthy individuals (Sun Y. et al., 2021). Notably, ITIH4 maybe as a promising potential diagnostic biomarker in the serum of patients with early gastric cancer (Sun Y. et al., 2021). Exosomes containing NNMT, which were derived from gastric cancer cells, have the capacity to facilitate peritoneal metastasis through the activation of the TGF-β/Smad2 signaling pathway (Zhu et al., 2021). Exosomes-derived T-cell co-stimulator and indoleamine 2,3-dioxygenase 1 levels in peripheral blood can predict the occurrence of immune-related adverse events in gastric cancer patients undergoing immunotherapy (Jiang et al., 2022). Serum exosomal Dicer could be considered as a potential non-invasive diagnostic biomarker for the early detection of differentiated gastric adenocarcinoma (Okuda et al., 2021).

Exosomal proteins play a pivotal role in the diagnosis and prognosis assessment of gastric cancer. They serve as potential biomarkers for early detection, enabling non-invasive screening methods that can identify the presence of gastric cancer (Table 1). Furthermore, specific exosomal proteins, such as PD-L1, can provide insights into the immune status of patients and predict their survival outcomes, thereby guiding prognostic evaluations and treatment strategies.

**TABLE 1:** Overview of exosomal proteins in gastric cancer diagnosis.

Protein	Source of exosomes	Roles	Isolation methods	References
DACT3-AS1	Cells exosomes	Diagnosis and treatment marker	Ultracentrifugation	Qu et al. (2023)
ILK1 and CD14	Peripheral blood exosomes and cells exosomes	Progression diagnostic markers	Commercial kits	Zhou et al. (2023)
DUOXA2, ITGA7, LIMS1, MSRB3, PLCB1, RAB6B, SEMA3C, SMTN, TADA1, TBC1D14	Patient serum exosomes	Diagnostic marker for peritoneal metastasis	Centrifugation	Chen et al. (2023)
HER2	Patient serum exosomes	Diagnosis and treatment marker	Commercial kits	Li et al. (2023a)
PD-L1	Patient serum exosomes and cells exosomes	Prognostic and diagnostic biomarker	Ultracentrifugation	Li et al. (2024b)
TRIM3	Patient serum exosomes and cells exosomes	Diagnostic marker	Ultracentrifugation	Fu et al. (2018)
Frizzled 10	Patient serum exosomes	Prognostic and diagnostic biomarker	Centrifugation	Scavo et al. (2021)
ITIH4	Patient serum exosomes	Diagnostic marker	Commercial kits	Sun et al. (2021a)
NNMT	Cells exosomes	Diagnostic marker for peritoneal metastasis	Ultracentrifuge using Commercial kits	Zhu et al. (2021)
EV-IDO1	Patient serum exosomes	Diagnostic marker of immune related adverse events	Use the SEC-based qEV column and centrifugation	Jiang et al. (2022)
Dicer	Patient serum exosomes	Diagnostic marker	Commercial kits	Okuda et al. (2021)

## 4 Roles of exosomal protein in gastric cancer progression

Exosomes play pivotal roles in gastric cancer progression through various mechanisms (Zhang et al., 2018; Wang et al., 2024; Liu et al., 2024; Zheng et al., 2018; Wu et al., 2023; W et al., 2022; Wu et al., 2022; Tsurusawa et al., 2022; Iha et al., 2022; Xue et al., 2020). They promote tumor cell detachment by carrying molecules related to cell adhesion and matrix degradation. Additionally, exosomes facilitate immune escape by carrying immunosuppressive molecules, thereby helping tumor cells evade immune surveillance. Exosomes also promote angiogenesis and pre-metastatic niche formation, creating a favorable environment for tumor cell colonization and metastasis. Furthermore, exosomes carry molecules that promote EMT, enhancing tumor cell migration and invasion capabilities.

The results of Zhang et al. demonstrated that exosomes derived from gastric cancer cells induce autophagy and promote pro-tumor activation of neutrophils through the HMGB1/TLR4/NF-κB signaling pathway (Zhang et al., 2018). These finding provided new insights on the mechanisms underlying neutrophil regulation in gastric cancer and highlights the multifaceted role of exosomes in modifying the tumor microenvironment. M2-polarized tumor-associated macrophages play an important role in promoting gastric cancer progression. These results of Wang et al. indicated that exosomes derived from M2-polarized tumor-associated macrophages contribute to the progression of gastric cancer through MALAT1-mediated regulation of glycolysis (Wang et al., 2024). Through its interaction with the transcription factor POU2F1, exosomal HMGB1 suppresses the transcriptional activity of p50, resulting in the inactivation of the NF-κB pathway (Liu et al., 2024). This, in turn, induced M2-like macrophage polarization and promoted the progression of gastric cancer (Liu et al., 2024). Zheng et al. found that apolipoprotein E was a highly specific and effective protein in M2 macrophage derived exosomes, and the transfer of apolipoprotein E to exosomes could promote the metastasis of gastric cancer cells (Zheng et al., 2018). The expression of RAB31 increased with gastric cancer progressed, and cells overexpressing RAB31 exhibited an enhanced capacity for migration (Wu et al., 2023). These results revealed a pivotal role for RAB31 in gastric cancer metastasis through the regulation of exosome secretion (Wu et al., 2023). It has been reported that overexpression of TOB1 induces autophagy in gastric cancer cells by secreting exosomes (W et al., 2022). Modified Jianpi Yangzheng has the capacity to decrease the level of exosomal PKM2 in gastric cancer cells. Importantly, modified Jianpi Yangzheng not only reduced the transfer of exosomal PKM2 from tumor cells to macrophages but also mitigated the differentiation of M2-TAM in the tumor microenvironment induced by exosomal PKM2(42). Angiogenesis plays a crucial role in gastric tumorigenesis, invasion, and metastasis by supplying essential oxygen and nutrients to the tumor. Tsurusawa et al. also found that exosomes-transferred GRP78 could promote the proliferation and migration of gastric cancer cells (Tsurusawa et al., 2022). Kanako et al. found that the exosomes GRP78 derived from gastric cancer cells enhanced angiogenesis after stimulating endothelial cells (Iha et al., 2022). YB-1, transferred via gastric cancer exosomes, promoted angiogenesis by augmenting the expression of angiogenic factors in vascular endothelial cells (Xue et al., 2020). Exosomal CD44 facilitates the transmission of lymph node metastatic potential between gastric cancer cells by inducing fatty acid oxidation reprogramming through a mechanism mediated by YAP and CPT1A (Wang et al., 2022). UBR2, which was enriched in exosomes derived from p53-deficient mouse bone marrow mesenchymal stem cells, promoted the progression of gastric cancer through activation of the Wnt/β-catenin signaling pathway (Mao et al., 2017). Lymph node metastasis-derived gastric cancer cells educate bone marrow-derived mesenchymal stem cells through the activation of YAP signaling by exosomal Wnt5a (Wang et al., 2021). This process may contribute to the progression and metastasis of gastric cancer (Wang et al., 2021). These data demonstrated that *H. pylori* infection-induced upregulation of MET in exosomes, which in turn educates tumor associated macrophages to promote gastric cancer progression (Che et al., 2018). Liu et al. found that the promotion of gastric carcinoma lymphatic metastasis by CD97 was dependent on exosomes (Liu et al., 2016). Exosomal PD-L1 promoted the establishment of an immunosuppressive microenvironment in gastric diffuse large B-cell lymphoma, thereby potentially impeding the immune system’s ability to combat the lymphoma (Zeng et al., 2023).

Exosomal proteins play a crucial role in the progression of gastric cancer. They act as key mediators in various biological processes. By influencing the tumor microenvironment and interacting with other cells, such as immune cells and surrounding normal cells, exosomal proteins contribute to the growth, migration and invasion of gastric cancer cells (Table 2). Exosomal proteins represent important targets for understanding the mechanisms underlying gastric cancer progression.

**TABLE 2:** Overview of exosomal protein in gastric cancer progression.

Protein/pathway	Source of exosomes	Target	Roles	Isolation methods	References
HMGB1	Cells exosomes	TLR4/NF-κB	Induce autophagy and promote pro-tumor activation of neutrophils	Ultrafiltration and ultracentrifugation	Zhang et al. (2018)
MALAT1	Cells exosomes	δ-catenin/HIF-1α	Promote gastric cancer progression *via* regulation of glycolysis	Ultrafiltration and ultracentrifugation	Wang et al. (2024)
HMGB1	Cells exosomes	p50/NF-κB	Induces M2-like macrophage polarization	Ultracentrifugation	Liu et al. (2024)
Apolipoprotein E	Cells exosomes	PI3K-Akt	Promote the migration of gastric cancer cells	Ultrafiltration and ultracentrifugation	Zheng et al. (2018)
RAB31	Cells exosomes	—	Increase tumor cell invasion and metastasis	Commercial kits	Wu et al. (2023)
TOB1	Cells exosomes	LC3-II/LC3-I	Induces autophagy	Differential ultracentrifugation	Wang et al. (2022)
PKM2	Cells exosomes	PI3K/Akt/mTOR	Induces M2 macrophages differentiation	Commercial kits	Wu et al. (2022)
GRP78	Cells exosomes	AKT	Enhance angiogenesis	Ultrafiltration and ultracentrifugation	Iha et al. (2022)
YB-1	Cells exosomes	VEGF, Ang-1, MMP-9 and IL-8	Enhance angiogenesis	Differential ultracentrifugation	Xue et al. (2020)
GRP78	Cells exosomes	—	Promote the cancer stemness	Ultrafiltration and ultracentrifugation	Tsurusawa et al. (2022)
CD44	Cells exosomes	YAP/CPT1A	Transmits lymph node metastatic capacity	Commercial kits	Wang et al. (2022)
UBR2	p53 deficient mouse exosomes	Wnt/β-catenin	Promote gastric cancer growth and metastasis	Commercial kits	Mao et al. (2017)
Wnt5a	Patient serum exosomes and cells exosomes	YAP	Enhance lymph node metastasis	Differential ultracentrifugation	Wang et al. (2021)
MET	Cells exosomes	IL-1β	Promote gastric cancer progression	Total exosome isolation reagent	Che et al. (2018)
CD97	Cells exosomes	CD55, CD44v6, α5β1, CD31	Promote lymphatic metastasis	Ultrafiltration and ultracentrifugation	Liu et al. (2016)
PD-L1	Patient serum exosomes and cells exosomes	CD20, CD79a, CD5, CD10, Ki67	Promotes the formation of an immunosuppressive microenvironment	Ultracentrifugation	Zeng et al. (2023)

## 5 Therapeutic potential of targeting exosomes in gastric cancer

Following the therapeutic and preventive roles of phytochemicals and natural ingredients in the treatment and prevention of tumors such as gastric cancer, exosomes and their carried proteins have demonstrated promising clinical application prospects (Khan et al., 2021; Ouy et al., 2021; Salama et al., 2022; Ding et al., 2021). Given the critical roles in gastric cancer progression, exosomes represent potential therapeutic targets. Targeting exosome-mediated signaling pathways or inhibiting exosome carrying pro gastric cancer molecules secretion could offer novel therapeutic strategies for gastric cancer. For instance, inhibiting the release or function of exosomes carrying immunosuppressive molecules could enhance the efficacy of immunotherapy. Additionally, targeting exosome-mediated drug resistance mechanisms could improve the response to chemotherapy.

Pretreatment plasma-derived soluble PD-L1 level could serve as a prognostic marker for patients undergoing cytotoxic chemotherapy. Meanwhile, serum-derived exosomal PD-L1 may indicate the immunosuppressive state of patients with advanced gastric cancer (Shin et al., 2023). It was reported that exosomal PD-L1 predicted poorer survival and reflects the immune status in patients with gastric cancer (Fan et al., 2019). Deletion of LSD1 leaded to a reduction in exosomal PD-L1 levels and restored T-cell responsiveness in gastric cancer (Shen et al., 2022). This discovery indicated a novel mechanism through which LSD1 may regulate cancer immunity in gastric cancer, thereby presenting a promising new target for immunotherapy against gastric cancer. It is reported that *Helicobacter pylori* CagA enhances the immune evasion of gastric cancer by upregulating the level of PD-L1 in exosomes. According to this finding, targeting CagA and exosomal PD-L1 can improve the immunotherapeutic efficacy of *H. pylori* infected gastric cancer (Wang J. et al., 2023). Melatonin has been found to enhance anti-tumor immunity by targeting the PD-L1 expressed on macrophages through exosomes derived from gastric cancer cells, suggesting a promising application of MLT in the realm of innovative anti-tumor immunotherapies (Wang K. et al., 2023). Sun and his colleagues discovered that eliminating exosomal PD-L1 may be a strategy to enhance the sensitivity of gastric cancer cells to PD-1 targeting therapy (Sun et al., 2024). The findings by Li et al. revealed that exosomal THBS1, originating from gastric cancer cells, augmented the functionality of Vγ9Vδ2 T cells through activation of the RIG-I-like pathway in an m6A methylation-dependent manner. Targeting the exosomal THBS1/m6A/RIG-I axis could have significant implications for the development of immunotherapy strategies against gastric cancer (Li J. et al., 2023). The results of Sun et al. showed that cisplatin-resistant gastric cancer cells enhance chemoresistance in cisplatin-sensitive cells through the exosomal RPS3-mediated PI3K/Akt/Cofilin-1 pathway (Sun M. Y. et al., 2021). GKN1 protein was secreted and internalized within the gastric epithelium through exosome-driven transfer mechanisms, exhibiting inhibitory effects on gastric tumorigenesis (Yoon et al., 2018). This characteristic supported the potential clinical application of GKN1 in the diagnosis and treatment of gastric cancer. The findings of Park et al. highlighted the promising potential of DE532 exosomes loaded with 17-DMAG as a potent therapeutic approach for gastric cancer, characterized by its precise targeting capability and the potential to significantly reduce adverse effects (Park et al., 2024). The exosome-mediated transfer of CLIC1 played an important role in promoting vincristine-resistance in gastric cancer (Zhao et al., 2019). Yoon and colleagues discovered that the exosomal GKN1 protein has an inhibitory effect on gastric carcinogenesis by downregulating the HRas/Raf/MEK/ERK signaling pathways (Yoon et al., 2020).

Exosomal proteins derived from gastric cancer cells and other cells play a significant role in the treatment of gastric cancer (Table 3). Exosomal proteins serve as potential therapeutic targets, offering novel avenues for the development of targeted therapies. By modulating the function of exosomal proteins, it may be possible to inhibit gastric cancer growth, block metastasis, and enhance the efficacy of immunotherapy. Furthermore, exosomes can be used as delivery vehicles for therapeutic agents, enabling more effective treatment of gastric cancer. Thus, the study of exosomal proteins has great potential in the treatment of gastric cancer.

**TABLE 3:** Overview of exosomal protein in gastric cancer treatment.

Protein	Source of exosomes	Target	Roles	Isolation methods	References
PD-L1	Patient serum exosomes	CD69 and PD-1	Predicted poorer survival and reflects the immune status in patients	Commercial kits	Shin et al. (2023)
PD-L1	Patient serum exosomes	MHC-I	Reflects the immune status in GC patients	Commercial kits	Fan et al. (2019)
PD-L1	Cells exosomes	LSD1	Restores T-cell response in gastric cancer	Differential ultracentrifugation	Shen et al. (2022)
PD-L1	Patient serum exosomes and cells exosomes	CagA	Promote immune evasion of gastric cancer	Commercial kits	Wang et al. (2023a)
PD-L1	Cells exosomes	MLT/TNF-α and CXCL10	Enhance anti-tumor immunity	Differential ultracentrifugation	Wang et al. (2023b)
PD-L1	Cells exosomes	CD63/PD1	Promote the PD-1 targeting therapy	Ultracentrifugation combined with sucrose density gradient centrifugation	Sun et al. (2024)
THBS1	Cells exosomes	m6A/RIG-I	Enhance immunotherapy	Differential ultracentrifugation	Li et al. (2023b)
RPS3	Cells exosomes	PI3K/Akt/Cofilin-1	Enhance chemoresistance	Ultracentrifugation	Sun et al. (2021b)
GKN1	Patient serum exosomes and cells exosomes	—	Inhibit gastric tumorigenesis	Centrifugation combined with separation column	Yoon et al. (2018)
DE532	Engineered exosomes	17-DMAG	Enhance efficacy of gastric cancer treatment	Centrifugation	Park et al. (2024)
CLIC1	Cells exosomes	P-gp/Bcl-2	Induce the development of resistance to vincristine	Ultracentrifugation	Zhao et al. (2019)
GKN1	Cells exosomes	HRas/Raf/MEK/ERK	ERK inhibitors	Centrifugation combined with separation column	Yoon et al. (2020)

## 6 Conclusion and future directions

### 6.1 Main functions and advantages of exosomes and their carrier proteins

Exosomes and their proteins have emerged as promising biomarkers and therapeutic targets in the diagnosis, occurrence, development, and treatment process of gastric cancer. Exosomes derived from gastric cancer cells carry substances that reflect the characteristics of the tumor cells, making them potential diagnostic biomarkers for gastric cancer. The protein expression profiles of exosomes often differ significantly across different types and stages of gastric cancer, indicating their close association with cancer development and progression. Exosomal proteins offer several advantages for cancer diagnosis: they are stable with long half-lives, can directly interact with target cells, and can be detected in smaller sample sizes with relatively simple separation procedures (Kalluri and LeBleu, 2020; Mathieu et al., 2021). Exosomes and their proteins also play crucial roles in various stages of gastric cancer progression, including immune regulation, tumor microenvironment, angiogenesis, EMT, invasion and metastasis. Exosomes have potential applications in cancer treatment, serving as drug delivery carriers or immunomodulators for tumor therapy. In addition, Exosomal proteins are gradually showing unique advantages as therapeutic targets for gastric cancer.

The bidirectional regulation of exosomal proteins in the tumor microenvironment is not only an accomplice to disease progression, but can also be engineered into a therapeutic weapon. For example, using gene editing technology to modify exosomes and their protein expression to carry targeted drugs or immune regulatory molecules. The possibility of developing personalized treatment strategies based on patient specific exosome characteristics, such as PD-L1 levels. Combined with immune checkpoint inhibitors, exosomal proteins may become a key indicator for predicting treatment response.

### 6.2 Problems and future challenges in clinical application

The complex mechanisms underlying exosome-mediated cancer progression are still not fully understood. The clinical application of exosomal proteins as diagnostic and therapeutic tools requires further validation and approval by regulatory agencies. Investigating the mechanisms underlying exosome-mediated gastric cancer occurrence and progression and to develop effective therapeutic strategies targeting exosomes. Developing novel exosome-based drug delivery systems for more effective cancer treatment. Exploring the potential of exosomal proteins as prognostic biomarkers and monitoring tools for treatment response. Addressing the ethical and regulatory challenges associated with the use of exosomes in clinical practice. Validating the clinical utility of exosomal proteins-based diagnostics and therapeutics in larger patient cohorts. Different extraction methods for exosomes may have an impact on the protein content they carry. Additionally, there may be significant variations in the detection of exosome-carried proteins as diagnostic biomarkers for gastric cancer using different protein detection methods. Therefore, in future clinical applications, we need to ensure the reproducibility and robustness of both exosome isolation and protein detection methods.

The differences in exosome isolation and detection methods may result in data being non reproducible. Suggest establishing international consensus guidelines and developing commercial test kits to promote clinical implementation. The heterogeneity of exosomes, such as functional differences in subgroups, may be underestimated. In the future, we may need to use “functional subtype classification” to understand the different roles of exosome and their protein heterogeneity in gastric cancer, and call for research on the unique roles of specific subgroups in gastric cancer. When exosomes are used as therapeutic carriers, it is necessary to address the issues of large-scale production and safety. It can be explored whether “synthetic exosomes” have clinical advantages over natural exosomes.

In conclusion, exosomes and their proteins hold great promise for improving the diagnosis, process and treatment of gastric cancer. With ongoing research and technological advancements, exosomal proteins could revolutionize the management of gastric cancer. By combining exosome research with cutting-edge technologies such as AI and nanomaterials, and focusing on clinical translation pain points, for example, the “Exosome Diagnosis 2.0 Era” requires a shift from single biomarkers to dynamic functional analysis, and treatment strategies should be upgraded from “blocking harmful exosomes” to “customizing beneficial exosomes.”
